# Horizontal MicroRNA Transfer by Platelets – Evidence and Implications

**DOI:** 10.3389/fphys.2021.678362

**Published:** 2021-06-03

**Authors:** Marion Mussbacher, Anita Pirabe, Laura Brunnthaler, Waltraud C. Schrottmaier, Alice Assinger

**Affiliations:** ^1^Department of Pharmacology and Toxicology, University of Graz, Graz, Austria; ^2^Center of Physiology and Pharmacology, Medical University of Vienna, Vienna, Austria

**Keywords:** microRNA, platelets, microvesicles, cellular communication, horizonal transfer

## Abstract

For decades, platelets have been known for their central role in hemostasis and their ability to release bioactive molecules, allowing inter-platelet communication and crosstalk with the immune system and vascular cells. However, with the detection of microRNAs in platelets and platelet-derived microvesicles (MVs), a new level of inter-cellular regulation was revealed. By shedding MVs from their plasma membrane, platelets are able to release functional microRNA complexes that are protected from plasma RNases. Upon contact with macrophages, endothelial cells and smooth muscle cells platelet microRNAs are rapidly internalized and fine-tune the functionality of the recipient cell by post-transcriptional reprogramming. Moreover, microRNA transfer by platelet MVs allows infiltration into tissues with limited cellular access such as solid tumors, thereby they not only modulate tumor progression but also provide a potential route for drug delivery. Understanding the precise mechanisms of horizontal transfer of platelet microRNAs under physiological and pathological conditions allows to design side-specific therapeutic (micro)RNA delivery systems. This review summarizes the current knowledge and the scientific evidence of horizontal microRNA transfer by platelets and platelet-derived MVs into vascular and non-vascular cells and its physiological consequences.

## Introduction

Platelets are anucleate, highly abundant blood cells that constantly patrol the microvasculature to monitor vascular injuries and act as sentinels to trace inflammatory and infectious processes. Thereby, platelets travel long distances and come in close proximity to various cell types, which allows inter-cellular communication and exchange of bioactive molecules. Apart from growth factors and immunomodulatory molecules, a broad repertoire of RNA species, including mRNAs, microRNAs (miRNA) and circular RNAs was detected in mouse and human platelets (Best et al., 2017). More precisely, platelets were reported to contain approx. 8500 mRNA and 500 miRNA species (Rowley et al., 2011), which are either inherited from their megakaryocyte precursors or taken up from plasma (Harrison and Goodall, 2008; Best et al., 2017; Gutmann et al., 2020). However, despite containing the complete translation machinery, only a handful of these mRNAs are *de novo* synthetized into proteins [e.g., interleukin (IL) 1β, B-cell lymphoma 3 protein (BCL3), tissue factor and coagulation factor XI] (Weyrich et al., 1998; Denis et al., 2005; Schwertz et al., 2006; Zucker et al., 2018). These low intraplatelet mRNA translation levels lead to the speculation that platelet miRNAs may fulfill functions beyond translation control, which rather depend on miRNA transfer to other cells than on platelet-restricted effects.

Belonging to the family of 19–24 nucleotide non-coding RNAs miRNAs mediate post-transcriptional gene regulation by sequence-specifically binding to the 3′ untranslated region (UTR) of mRNAs, thereby repressing translation and/or destabilizing mRNAs. Nowadays, miRNAs are predicted to regulate about 60% of human genes and horizontal transfer of mRNA might represent a mechanism for inter-cellular communication and fine-tuning of the microenvironment. Due to their unique expression patterns, which mirror cellular (im)balances, miRNAs bear enormous biomarker potential as they are suggested to serve as “fingerprints” of various diseases. Platelets express the complete miRNA machinery including ribonuclease III Dicer, RNA-binding protein 2 and Argonaute 2 (Ago2), which allows processing of pre-miRNA into mature miRNA and formation of functionally competent Ago2-miRNA effector complexes (Landry et al., 2009). The lack of genomic DNA and the inability to synthetize new miRNAs, renders platelets an ideal cellular tool to study miRNA transfer to nucleated recipient cells. Moreover, as a consequence of their origin from megakaryocytes, platelets exhibit a unique cellular structure with an open canalicular system (OCS), a dense tubular system (DTS) and several types of granules, which not only protect circulating miRNAs from degradation from plasma RNases, but also allow their regulated, site-specific delivery.

A prerequisite for efficient miRNA transfer is close proximity between donor and recipient cells, which is facilitated by a broad variety of platelet surface molecules such as P-selectin, glycoprotein (GP)Ib, GPIIb/IIIa, intercellular adhesion molecule (ICAM) 2 and CD40 ligand (CD40L), which are exposed on the platelet surface upon activation. This pairing of miRNA transfer to platelet activation, guarantees a concerted and fine-regulated – however not fully deciphered – release of cellular material at specialized loci within the human body. Besides degranulation, activated platelets shed microvesicles (PMVs) with an approximate size of 100 nm to 1 μm from their plasma membrane into the extracellular space. These PMVs – first described in 1967 as “platelet dust” – are enriched for miRNAs and express high phosphatidylserine (PS) concentration on their outer membrane (Aatonen et al., 2012). In plasma of healthy individuals 70–90% of extracellular vesicles are of platelet origin, and they enable vesicle-mediated delivery of signaling complexes, RNA species and transcription factors that have the potential for epigenetic reprogramming of their recipient cells. PMVs are increased in various diseases such as cancer and immune disease and due to their small size allow the transport of genetic information into specialized microenvironments such as permeable vasculature of solid tumors.

## General Delivery Mechanisms

**FIGURE 1:**
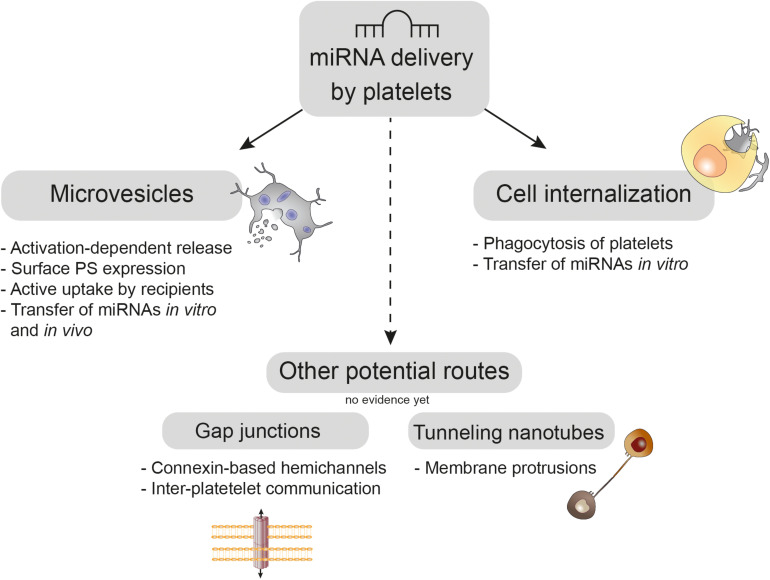
Platelet-mediated delivery of miRNAs. Upon activation platelets shed phosphatidylserine (PS)-rich microvesicles from their budding cell membrane, which are actively taken up by recipient cells *in vitro* and *in vivo*. Moreover, platelets can be directly internalized by recipient cell phagocytosis, which was demonstrated *in vitro*. Whereas platelets use connexin-based gap junctions for inter-platelet communication, the role of these hemichannels in platelet miRNA transfer needs to be elucidated. Tunneling nanotubes are long membrane protrusions that enable exchange of nucleic acids between cells; however, their usage for platelet-mediated miRNA transfer has not been shown, yet.

Several potential routes for miRNA transfer by platelets have been discussed, which either depend on direct heterotypic cell interaction via connexin-based gap junctions or tunneling nanotubes (TNTs) or on uptake of platelets and PMVs by recipient cells. An overview of these mechanisms is given in Figure 1.

### Microvesicles

Microvesicles (MVs) are small, spherical-like extracellular vesicles that are shed from the budding plasma membrane of activated cells (Lu et al., 2017). Their size of 100 nm–1 μm distinguishes MVs from exosomes and apoptotic bodies, which have a diameter of 40–100 nm and 1–5 μm, respectively (Kalra et al., 2012; Lv et al., 2019). MVs are composed of a phospholipid (PL) bilayer and retain cellular components of their parent cell such as proteins (e.g., surface molecules), lipids and nucleic acids. MV release can be triggered by cell activation, shear stress, hypoxia, apoptosis and prolonged storage (Vion et al., 2013; Berezin et al., 2016; Ridger et al., 2017), and involves reconstruction of the plasma membrane: In resting cells stability of the PL bilayer is mediated by inward (flippases), outward (floppases), and bi-directional (scramblases) transporters (Ridger et al., 2017). Disruption of membrane stability due to calcium-mediated inhibition of flippase translocates PS from the inner to the outer leaflet (Bevers and Williamson, 2016) and causes MV shedding into the extracellular space (Ridger et al., 2017). MVs can travel long distances before being taken up by recipient cells (Maas et al., 2017). MV internalization is thought to be mediated by endocytosis, phagocytosis and membrane fusion (Parolini et al., 2009; Feng et al., 2010; Svensson et al., 2013). These complex cellular processes are supported by a plethora of molecules, including selectins, PS, lactadherin, β2-GP I, and developmental endothelial locus-1 (Falati et al., 2003; Dasgupta et al., 2009, 2012; Abdel-Monem et al., 2010; Kirschbaum et al., 2015). PS exposure acts as “eat me” signal for phagocytic cells, however, MVs are also opsonized by the complement component C3b and subsequently bind to complement receptor 1 (CR1) (Flaumenhaft, 2006). Moreover, MV internalization is an active, highly regulated process that involves the concerted actions of several enzymes and thereby limits MV internalization to specific microenvironments: In rheumatoid arthritis, for example, the presence of both secreted phospholipase A2 IIA (PLA2IIA) and PMV-derived 12-lipoxygenase is necessary to enable MV internalization by neutrophils (Duchez et al., 2015). A better understanding of the regulation of selective MV uptake mechanisms might reveal specific processes that promote the exchange of genetic material.

Microvesicle release has been reported for different cell types of the vascular system including endothelial cells, smooth muscle cells, red blood cells, leukocytes, and platelets (Burger et al., 2013; Liu et al., 2018) and MVs can therefore be detected in various body fluids such as blood, salvia, synovial fluid and urine (Zmigrodzka et al., 2016). With an estimated concentration of 10^4^ MV/mL PMVs represent the most abundant MV population in peripheral blood of healthy individuals (Laffont et al., 2013; Boilard et al., 2015; Zmigrodzka et al., 2016). However, since platelets and their megakaryocyte precursors share the same surface receptors (e.g., CD41 and CD61) (Berckmans et al., 2001; Gitz et al., 2014), it is difficult to differentiate between MVs that are released from bone marrow-resident megakaryocytes during platelet production and MVs that are shed from platelets during activation (Flaumenhaft et al., 2009; Owens Mackman, 2011; Gitz et al., 2014). Some studies propose that only platelet-derived MVs but not megakaryocyte-derived MVs are elevated under pathological conditions and contribute to inflammatory and cardiovascular diseases (Forlow et al., 2000; Arraud et al., 2014). In this context, complement proteins (e.g., C5b-9), bacterial lipopolysaccharide (LPS) and viruses induce PMV shedding (Sims et al., 1988; Flaumenhaft et al., 2009; Gitz et al., 2014; Boilard et al., 2015) and PMV-mediated transfer of miRNAs was associated with cardiovascular diseases (Gidlof et al., 2013; Tan et al., 2016), immune responses and tumor biology (Laffont et al., 2013, 2016; Liang et al., 2015). Besides their important transport function, PMVs provide a procoagulant surface (Tans et al., 1991; Tan et al., 2016), which facilitates binding of coagulation factors FVIII, FVa, FXa, FII, and FX to support thrombin generation (Sims et al., 1988; Heijnen et al., 1999; Owens Mackman, 2011; Frisch et al., 2019).

### Cellular Internalization

Genetic material can also be transported by the uptake of whole cells (e.g., platelets) by recipient cells such as monocytes, macrophages, neutrophils, endothelial cells and smooth muscle cells (Badlou et al., 2006; Badrnya et al., 2014; Daito et al., 2014; Zeng et al., 2019; Ji et al., 2020). In this context, platelet internalization represents a mode of communication beyond the sole removal of aged platelets from the circulation (Berger et al., 1998; De Meyer et al., 2002) despite its seemingly unrefined nature. During phagocytosis, platelet receptors, chemokines, bioactive lipids, and nucleic acids get transferred, which can attenuate cell apoptosis and promote survival and proliferation of the recipient cell (Jiang et al., 2015; Zeng et al., 2019). Recent literature indicates that the regulatory potential of platelet internalization may vary between different recipient cells and depends on the (patho)physiological context (Zeng et al., 2019).

## Potential Delivery Systems

### Gap Junctions

Gap junctions connect homo- and heterotypic adjacent cells, enabling intercellular communication and bidirectional exchange of small molecules (1–1.8 kDa), such as ions, metabolites, second messengers and peptides (Neijssen et al., 2007; Figueroa and Duling, 2009; Goodenough and Paul, 2009). Typically, gap junctions are formed by adherent cells such as endothelial cells and vascular smooth muscle cells (Payne et al., 2004; Isakson and Duling, 2005), however, gap junction-mediated transfer has also been observed for circulating cells such as monocytes (Neijssen et al., 2007) and platelets (Angelillo-Scherrer et al., 2011; Vaiyapuri et al., 2012). Gap junctions are formed in the cell membrane by oligomerization of six connexin monomers, which build up individual hemichannels. The connection of hemichannels of neighboring cells forms a pore across the extracellular space with a mean pore diameter of 2 nm (Goodenough and Paul, 2009; Koval et al., 2014). This pore enables passage of bioactive material and has been attributed an important role in various vascular and inflammatory diseases including atherosclerosis (Pfenniger et al., 2013), hypertension (Looft-Wilson et al., 2012), and diabetes (Serre-Beinier et al., 2000). To date, the gap junction-mediated transport of miRNAs has been investigated in various cell types (Zong et al., 2016; Lemcke et al., 2017). These studies demonstrate that functional miRNAs are transferred by gap junctions, however the efficiency of the transmission depends on the specific involved connexin proteins and might be limited by the size of the miRNAs (approx. 7 kDa). So far, no studies on platelet-mediated transport of miRNAs via gap junctions have been reported.

### Tunneling Nanotubes

Tunneling nanotubes (TNTs), also termed membrane nanotubes, are long, thin membrane protrusions, which connect the cytoplasm of neighboring cells (Korenkova et al., 2020). These structures efficiently transport a wide variety of cargos, including organelles, vesicles (Rustom et al., 2004; Onfelt et al., 2006; Mineo et al., 2012), plasma membrane components, cytoplasmic molecules and even pathogens (Rustom et al., 2004; Onfelt et al., 2006; Sowinski et al., 2008; Kumar et al., 2017). TNTs are built from non-adherent filamentous actin (F-actin) fibers and are usually over 100 μm long and 50–500 nm in diameter (Sartori-Rupp et al., 2019). TNTs comprise of several individual tunneling nanotubes (iTNTs) that run in parallel and are connected by N-cadherins. Extension of these iTNTs between adjacent cells thus forms TNTs. TNTs have been observed in several non-moving cells (Schiller et al., 2013; Halasz et al., 2018; Panasiuk et al., 2018; Li et al., 2019) and have gained attention in immunology, neurobiology and especially tumor biology (Schiller et al., 2013; Lu et al., 2019; Sartori-Rupp et al., 2019). While TNTs can facilitate tumor growth and the spread of pathogens, TNT mediated communication during acute inflammation promotes a rapid and coordinated response of immune cells e.g., via delivering antigens (Zaccard et al., 2016; Halasz et al., 2018; Panasiuk et al., 2018). However, although TNT-mediated transmission of nucleic acids and miRNAs between adjacent cells has been reported (Haimovich et al., 2017, 2020), it is currently unknown if platelets employ this mechanism to communicate with adjacent cells.

## Horizontal Gene Transfer

The aforementioned delivery mechanisms can be employed to transport miRNAs from one cell of an organism to another one in a process that is classified as horizontal gene transfer (HGT). HGT is the lateral exchange of genetic material between different species and was first described in bacteria, which adapt to changes in their microenvironment by transfer of genetic material (Redis et al., 2012). In contrast to vertical gene transfer which occurs between cellular generations (Soucy et al., 2015), HGT has evolved as a rapid method to acquire new cellular functions by inserting sequences or entire genes derived from different species (Keeling and Palmer, 2008). Recent studies indicate that HGT may also occur in multicellular organisms and secreted miRNAs mediate genetic – but not genomic – adaptations to (micro)environmental changes. Extracellular miRNAs in various body fluids such as plasma, salvia and breast milk shuttle between different cell types and mediate horizontal cell-cell communication over long distances (Chim et al., 2008; Cheng, 2015). So-called circulatory miRNAs are protected from degradation by highly abundant, endogenous RNases *via* enclosure into MVs, lipoproteins [e.g., high density lipoprotein (HDL)], or via complex formation with RNA-binding proteins such as nucleophosmin 1 or Ago2 (Cui et al., 2019). Circulatory miRNAs bear the potential to fine-tune recipient cell functions and are therefore potentially involved in the progression of various diseases, such as cancer (Hu et al., 2012).

## Evidence on Horizontal miRNA Transfer by Platelets

**FIGURE 2:**
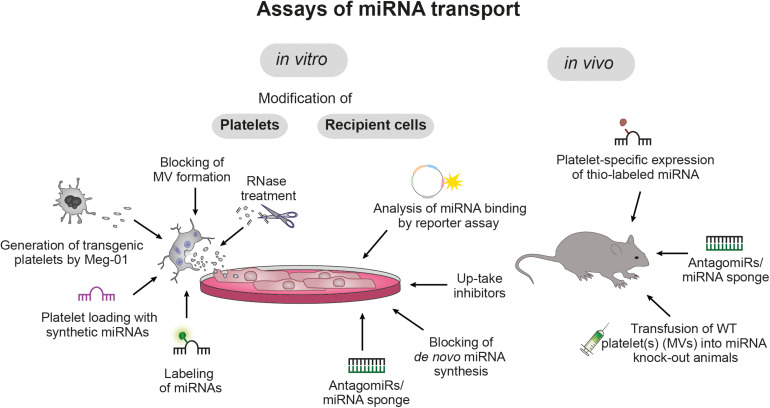
Assays of miRNA transport. Currently, platelet-mediated transfer of miRNAs is studied *in vitro* and *in vivo* with various molecular and genetic tools that allow targeting of platelets, recipient cells and miRNAs: Platelets can be loaded with synthetic or labeled miRNAs, microvesicle (MV) release can be blocked with brefeldin A and platelet RNA can be degraded via RNase treatment. Moreover, the megakaryoblast cell line Meg-01 allows *in vitro* production of genetically modified platelets. In recipient cells, uptake of platelet-derived miRNAs can be blocked by specific inhibitors (e.g., targeting of PLA2IIA) and binding of miRNAs to the recipient cell RNA can be prevented by co-incubation with short target site-specific anti-sense antagomiRs or miRNA sponges, which harbor multiple miRNA target sites. Furthermore, blocking *de novo* miRNA synthesis in recipient cells allows to distinguish between endogenous and transferred miRNAs and the inhibitory potential of miRNAs can be quantified by reporter assays. *In vivo* studies take advantage of genetically or pharmacologically modified mice to study *in vivo* miRNA transfer.

The high abundancy of platelets in the circulation, their sensitive nature, their manifold receptors and their potential to release bioactive molecules in a concerted, activation-dependent manner, renders platelets the ideal cells to accomplish HGT. Although internalization of whole platelets and PMV by other cell types such as endothelial cells, macrophages and hepatocytes has long been recognized as biological process, only recently (simultaneous) transfer of genetic material (e.g., miRNAs) and its consequences on the transcriptome and functionality of the nucleated recipient cells gained attention. While many aspects remain to be deciphered, an overview of current tools to study horizontal miRNA transfer is given in Figure 2 and the current knowledge on platelet-mediated miRNA transfer on vasculature, immune system and cancer development is summarized below.

### Horizontal miRNA Transfer by Platelets and Its Effects on Vasculature

First evidence of platelet mediated miRNA transfer derives from the co-culture experiments of monocyte-like THP-1 cells and human umbilical vein endothelial cells (HUVECs) with platelet-like particles (PLPs) derived from the megakaryoblastic cell line Meg-01 (Takeuchi et al., 1998). PLPs resemble the characteristics of human platelets and transfer labeled RNA to the vascular recipient cells. Despite being a mix between platelet and PMV, PLPs have the advantage of being easily modified *in vitro:* By transfecting Meg-01 cells with vectors such as GFP or siRNAs, genetically modified platelets can be generated. Although the miRNA repertoire of platelets, PLPs, and PMVs might differ in its quantity and quality, it is likely that general delivery and regulatory mechanisms are similar. Internalization of PLPs depends on the activation state of the recipient cells, yielding in successful transfer of platelet RNA to approx. 20% of recipient cells. The functionality of these transferred RNAs was demonstrated by loading of PLPs with an artificial GFP vector, which causes green fluorescence in recipient cells, which remained after PLP removal, while the signal decreased upon RNase treatment of PLPs (Risitano et al., 2012). These *in vitro* findings were validated *in vivo* by transfusion of wild-type platelets into TLR2-deficient mice followed by injection of LPS, which increased *in vivo* platelet-leukocyte interaction. After 6 h an increase in TLR2 mRNA in PBMCs could be detected, indicating successful transfer of genetic material between platelets and leukocytes *in vivo* (Risitano et al., 2012).

Further studies investigated whether the proliferative effect of PLPs on the hepatocellular carcinoma/hepatoblastoma cell line HepG2 was regulated by PLP internalization and subsequent RNA transfer, which would be of high relevance for liver regeneration (Kirschbaum et al., 2015): PLPs are quickly internalized by HepG2 and detected in close proximity to the nucleus and the endoplasmic reticulum. Interestingly, PLP internalization occurred in over 50% of co-incubated HepG2 cells and was also observed *in vivo* in regenerating liver tissue (Kirschbaum et al., 2015). Mechanistically, internalization of PLPs is independent of GPIb-dependent clearance of aged and dysfunctional platelets by hepatocytic Ashwell-Morell receptors, but crucially involves PS (Kirschbaum et al., 2015). Functionality of this mechanism was verified by transfer of fluorescence-labeled mRNA from PLPs to HepG2 cells, causing a significant increase in fluorescence signal in recipient cells, which was partially prevented by incubation of PLPs with RNA-degrading enzymes (Kirschbaum et al., 2015). Although Meg-01-generated PLPs might differ from native platelets regarding their receptor repertoire and RNA machinery, these studies clearly demonstrate the possibility for horizontal transfer of nucleic acids by platelets.

The most abundant miRNA in human platelets is miR-223 which is released upon thrombin-mediated platelet activation, preferentially within MVs (Laffont et al., 2013). Co-incubation of HUVECs with MVs from activated but not resting platelets caused an increase of miRNA-223 in HUVECs that was associated with uptake of MVs into the endothelial cytosol, thus demonstrating successful HGT of miRNA. Moreover, platelet MVs (PMVs) contained functional miR-223-Ago effector complexes, which were necessary for translational repression by miRNAs and suppressed the expression of two endogenous target genes at both mRNA and protein levels. This effect of platelet-derived miR-223 was prevented by introducing a miR-223 blocking sequence (known as miRNA sponge) into the recipient cell (Laffont et al., 2013). Whereas the functional consequences of PMV-mediated miR-223 delivery are not yet fully elucidated, insulin-like growth factor 2 receptor was identified as an endothelial target of miR-223, which was significantly decreased upon incubation of HUVECs with PMV (Pan et al., 2014). Moreover, presence of PMVs increased endothelial apoptosis in a miR-223-dependent manner upon incubation with advanced glycation end products (Pan et al., 2014).

Besides miR-223, miRNA Let-7a was found to be highly abundant in PMVs and was associated with the pro-angiogenic potential of PMV (Anene et al., 2018): Co-incubation of PMVs with HUVECs led to active transfer of miRNA Let-7a and subsequent translational repression of the anti-angiogenic molecule thrombospondin-1 (TSP-1) by targeting its mRNA.

The broad expression of miRNAs by various cell types may represent a hurdle to obtain solid verification of platelet miRNA transfer. To overcome this obstacle and provide undisputable proof, platelets were transfected with the synthetic exogenous miRNA syn-cel-miR-39, which is released upon platelet activation (Gidlof et al., 2013). Co-incubation with activated but not resting platelets led to a strong increase of Syn-cel-miR-39 in endothelial cells (Gidlof et al., 2013). In line, transfection of platelets with fluorescence-labeled scramble miRNA visually demonstrated the uptake of labeled PMVs into recipient cells and their subsequent presence in the cytosol. Transfer of miRNA was dependent on platelet activation and inhibited by the MV release inhibitor Brefeldin A, which blocks guanine nucleotide-exchange protein BIG2 (Islam et al., 2007). These observations have been validated for miR-22, miR-185, miR-320b, and miR423-5p, which were identified by a miRNA screening of platelets derived from myocardial infarct patients (Gidlof et al., 2013). Blocking miR-320b in endothelial cells caused an upregulation of ICAM-1, which was rescued upon incubation with platelet releasates (Gidlof et al., 2013). Moreover, stimulation of human platelets with an immune complex revealed the release of PMVs containing miR-96 and miR-26a (Hu et al., 2018). Transfection of platelets with miR-96 and miR-26a mimics followed by coincubation with HUVECs inhibited wound healing and vesicular network formation *in vitro*, which was associated with decreased expression levels of *SELP* and *PDGFRA* (Hu et al., 2018).

Besides endothelial cells, smooth muscle cells (SMCs) were identified as potential recipient cells for PMV-mediated miRNA (Tan et al., 2016): Incubation of miR-223-, miR-339-, and miR-21-containing exosomes derived from thrombin-stimulated platelets were shown to downregulate PDGFRβ in SMCs and inhibit their proliferation *in vitro*.

### Horizontal MicroRNA Transfer by Platelets and Its Effects on Immune Cells and Hematopoiesis

In addition to the evidence on the transfer of platelet TLR2 mRNA to PBMCs *in vivo* (Risitano et al., 2012), other experiments focused on the delivery of miRNA to leukocytes:

Co-incubation of fluorescently labeled PMV with primary human macrophages led to PMV internalization and subsequent enrichment of miR-126-3p in the recipient macrophage independently of *de novo* transcription (Laffont et al., 2016). Using bioinformatics approaches, *ATF3*, *ATP1B1*, *ATP9A*, and *RAI14* were identified as potential miRNA targets and their miR-126-3p-dependent downregulation was verified on mRNA level. Moreover, transcriptome-wide microarray analysis revealed additional upregulation of 34 miRNAs and a concomitant downregulation of 367 RNAs in macrophages upon incubation with PMVs. Among the identified targets of PMVs in macrophages, cytokines and chemokines such as CCL4, CSF1, and TNF-α were significantly downregulated. While it is unclear, which miRNAs are involved in the cellular reprogramming of macrophages, co-incubation of PMVs led to an increase in phagocytosis, pointing toward a potential role of PMV in shaping macrophage functions (Laffont et al., 2016). Further studies are warranted to differentiate between direct effects of platelet-mediated miRNA transfer and indirect effects of *de novo* transcription in macrophages.

An important role for PMV-derived miRNAs in the regulation of thrombopoiesis during increased platelet consumption and replenishment was identified in a mouse model of carbon tetrachloride-induced liver injury. In this paper, injection of PMV, which were highly enriched in miR-1915-39, led to TPO-independent increase of megakaryocytes and platelets by suppressing a member of the Rho GTPase family in hematopoietic stem cells (Qu et al., 2020).

### Horizontal MicroRNA Transfer by Platelets and Its Effects on Cancer

Accumulating evidence suggests that secretion of miRNAs by cancer cells mediates intercellular crosstalk during different stages of tumorigenesis and metastasis in a hormone-like fashion (Skog et al., 2008; Milman et al., 2019) and that exchange of miRNAs between different cell types (e.g., cancer cells, mesenchymal stem cells, and vascular endothelial cells) represents a powerful tool to shape microenvironments in a precisely regulated manner.

Horizontal miRNA transfer by platelets is therefore also most intensively studied in tumor models. Due to the rapid growth of solid tumors, the tumor neovasculature is perforated and highly permeable, allowing interaction of tumor cells with blood-derived components such as PMV, thereby opening the possibility for miRNA transfer: Indeed, PMVs infiltrate human and mouse tumors *in vitro* and *in vivo.* Daily infusion of PMVs inhibited growth of lung and colon carcinomas, which was prevented by inhibition of miR-24 by antagomiRs (Michael et al., 2017). In line with the anti-proliferative effects of PMV, knockout of the thrombin receptor PAR4, which results in decreased endogenous PMV levels, accelerated tumor growth in the absence, but not in presence of injected PMV. Mitochondrial mt-Nd2 and Snora75 RNAs are targeted by miR-24, which caused mitochondrial dysfunction and growth inhibition in tumor cells (Michael et al., 2017). To assure that alterations in solid tumor miRNAs are indeed a consequence of active miRNA transfer from PMVs and not a consequence of tumor cell *de novo* transcription, endogenous platelet RNA was genetically labeled by platelet/megakaryocyte-specific expression of uracil phosphoribosyltransferase, leading to thio-RNAs upon injection of 4-thiouracil. Presence of thio-miR-24 in solid tumor provided direct evidence for PMV-mediated miRNA transfer *in vivo* in the absence of any artificial transfection (Michael et al., 2017).

In a different cancer type, non-small cell lung cancer (NSCLC), PMV-mediated transfer of miR-223 promoted tumor invasiveness of human lung cancer cells (A549) by targeting the cytoskeletal protein erythrocyte membrane protein band 4.1-like 3 (EPB41L3) (Liang et al., 2015). PMV co-incubation with A549 cells led to miR-223 increase in the recipient cell, which was not associated with elevated tumor cell pre-miR-223s. Transfection of A549 cells with EPB41L3 siRNA or miR-223 mimic recapitulated the effect of PMV on tumor invasiveness. Interestingly, uptake of fluorescent PMVs into A549 recipient cells only occurred at 37°C and not at 4°C, indicating an active internalization process. However, the precise uptake mechanism and its regulation remains unclear.

In a screening assay to investigate factors associated with the malignant behavior of epithelial ovarian cancer (EOC), PMVs were identified to increase proliferation, migration and epithelial-mesenchymal transition of SKOV3 cells (Tang et al., 2017). Comparison of PMVs from thrombin-stimulated platelets and apoptotic platelets identified miR-939 as potential mediator of tumor proliferation. Uptake of PMVs by SKOV3 cells could be blocked by knockdown of PLA2IIA, indicating a potential mechanism for platelet-cancer cell crosstalk, which helps to understand the association of thrombosis with poor EOC prognosis.

In summary, both pro- and anti-proliferative effects of platelet-derived miRNAs could be identified in various pathophysiologic states. These discrepancies may arise from different PMV preparations (e.g., different centrifugation protocols), sources (e.g., *in vitro* generated PMV versus PMV isolated from plasma) and quantities of incubated/injected PMVs. Moreover, dependent on the type and state of the disease and cell types involved, platelet-derived miRNAs might exert different roles.

## Potential Use of Platelets as Drug Delivering Agents

Recently, platelets have garnered increasing interest in cell engineering for platelet-based drug delivery that takes advantage of their unique characteristics. In particular, their lifespan of 7–10 days, their ability to home to sites of injury, inflammation or tumorigenesis and their rapid responses upon vascular or immunologic insults make platelets optimal tools for site-directed and controlled release of therapeutics superior to nanoparticle systems such as liposomes (Lu et al., 2018). Indeed, platelets coupled with anti-PD-L1 release the therapeutic antibody upon activation, presumably via dissociation or release of PMVs, thereby facilitating anti-PD-L1 transport and ensuring effective targeting of remaining cancer cells in a murine model of tumor ablation (Han et al., 2019). In a murine leukemia model the tumor-homing capacity of platelets was further enhanced by covalently linking anti-PD-1 conjugated platelets with hematopoietic stem cells, which ensured prolonged bioavailability and high therapeutic efficacy (Hu et al., 2018). Furthermore, in a murine lymphoma model platelet-loading with doxorubicin prolonged plasma presence of the chemotherapeutic and led to its enrichment specifically in tumor tissue, thereby reducing adverse effects and increasing the therapeutic efficacy (Xu et al., 2017a). This effect was even more pronounced when doxorubicin-loaded platelets were conjugated to an anti-CD22 antibody which facilitates internalization via the endocytic CD22 receptor (Xu et al., 2017b). Alternatively, platelets can be loaded with liposome-encapsulated physiologic substances that cannot be directly injected to enhance endogenous responses. For instance, liposomal thrombin can be endocytosed by platelets, thereby improving their coagulability *in vitro* (Chan et al., 2018).

In a different approach, a patient’s platelets may be hybridized to synthetic capsules that contain therapeutics and burst upon platelet activation due to the physical contractions occurring during platelet shape change. *In vitro* experiments using FVIII as hitchhiking capsule load have proven that this approach shields the therapeutic from circulating inhibitors until delivery, enables a targeted burst release and thus also enhances the therapeutic efficacy (Hansen et al., 2017).

Although the use of platelets as drug delivery tools in clinical settings may seem like a utopian idea in its infancy, successful *in vitro* and *in vivo* tests have proven that platelet-inspired therapy may not be as far-fetched after all, even though some obstacles still remain (Kola et al., 2021). However, the feasibility of platelet-based site-directed delivery of miRNAs as a means to interfere with pathological processes remains to be investigated. While not all mechanistic approaches may be usable for targeted transport and release of miRNAs, delivery via liposomal encapsulation or PMVs bear great therapeutic potential. After all, as presented above, PMVs can deliver miRNAs *in vitro* and *in vivo*, triggering functional changes of target cells (Liang et al., 2015; Laffont et al., 2016).

## miRNAs as Biomarkers and Therapeutic Agents

High stability and abundant presence of miRNAs in various body fluids in combination with their disease-specific characteristics point toward high theragnostic potential of miRNAs. Multiple studies have proven that circulating miRNAs represent powerful, easily accessible diagnostic tools, allowing prediction of disease progression, survival and therapeutic response of patients (Starlinger et al., 2019; Condrat et al., 2020). However, quality control and standardized sampling techniques are necessary to guarantee compatibility of samples between patients and studies (Valihrach et al., 2020). As platelets are an important source of circulating miRNAs (Sunderland et al., 2017), optimized anticoagulation is necessary to avoid artificial platelet activation (Mussbacher et al., 2017) and subsequent miRNA release (Mussbacher et al., 2020).

Moreover, miRNA-based therapies already entered the first clinical trials and both miRNA mimics and antagomiRs revealed high potential for the treatment of various types of cancers. Whereas the current potential therapeutic miRNA candidates are summarized elsewhere (Forterre et al., 2020), miR-34 mimics encapsulated in liposomal nanoparticles (MRX34) (Beg et al., 2017), miR-16 mimic (MesomiR-1) loaded in bacteria-derived nanocells (Reid et al., 2016), and anti-miR-155 (MRG-106) (Rupaimoole et al., 2016) are mentioned here as they represent examples of the first steps toward the clinical use of miRNA-based therapeutics.

## Outlook

Although it is clear that platelet/PMV-mediated miRNA transfer is a powerful tool to modulate cellular functions in a broad range of diseases, many questions remain unanswered and further research is warranted to unravel the full potential and limitations of horizontal miRNA transfer by platelets. One remaining question concerns the mechanisms of megakaryocyte-mediated miRNA packing and its adaptation to pathologic condition (e.g., to chronic inflammation). As most studies focus on the transfer of miRNAs from healthy platelets or transfected platelets, little is known on miRNA transfer of disease-educated platelets to their respective recipient cells although disease states might impact these transport mechanisms. Further, platelets potentially selectively take up certain miRNAs and may act as a liaison of miRNA-communication between different cell types – independently of their own miRNA transcriptome. Whereas most studies focus on the PMV uptake, mechanisms of intracellular miRNA liberation as well as their activation-dependent extracellular release under different conditions still need to be deciphered.

## Author Contributions

MM, LB, AP, and WS: writing – original draft preparation. AA: writing – review and editing. All authors contributed to the article and approved the submitted version.

## Conflict of Interest

The authors declare that the research was conducted in the absence of any commercial or financial relationships that could be construed as a potential conflict of interest.
